# Obstructive Infantile Hydrocephalus Secondary to Meningoventriculitis: A Case Report

**DOI:** 10.31729/jnma.8219

**Published:** 2023-07-30

**Authors:** Rupesh Raut, Shailes Paudel, Prakriti Bhandari, Umang Gupta, Anil Nepali

**Affiliations:** 1 Department of Neurosurgery, Patan Academy of Health Science, Lagankhel, Lalitpur, Nepal; 2 Department of Intensive Care Unit, Patan Academy of Health Sciences, Lagankhel, Lalitpur, Nepal; 3 Badegaun Primary Health Care Center, Badegaun, Lalitpur, Nepal; 4 Department of Emergency Medicine, Maharajgunj Medical Campus, Maharajgunj, Kathmandu, Nepal

**Keywords:** *case reports*, *hydrocephalus*, *infectious ventriculitis*, *meningitis*, *ventriculoperitoneal shunt*

## Abstract

Obstructive infantile hydrocephalus may arise due to anatomic or functional obstruction of cerebrospinal fluid flow. Obstruction of the aqueduct of sylvius (aqueductal stenosis) causes dilation of the lateral and third ventricles, while the size of the fourth ventricle remains relatively normal. Obstructive infantile hydrocephalus with meningoventriculitis is a rare phenomenon, and literature with only 2 other children with similar findings have been reported. We hereby report a case of a 16-week-old infant who developed *Escherichia coli* meningoventriculitis, later complicated by the development of hydrocephalus, challenging the management. The diagnosis was based on the magnetic resonance imaging of the brain, which showed hydrocephalus, and the cerebrospinal fluid culture showing *Escherichia coli* meningoventriculitis. The case was managed with serial ventricular drainage along with antibiotics followed by staged ventriculoperitoneal shunting. Serial measurement of head circumference is essential to prompt diagnostic suspicion in the case of paediatric meningitis.

## INTRODUCTION

Any obstruction to the cerebrospinal fluid flow that causes the ventricles to expand, results in hydrocephalus.^1^ Infantile hydrocephalus can coexist with a number of developmental disorders that have an embryological or fetal basis.^2^ An infant with hydrocephalus will typically have a head circumference that enlarges at an abnormal rate, which distinguishes hydrocephalus from macrocrania.^3^ Meningitis is usually related to communicating hydrocephalus, unlike our case, where features of obstruction were developed. Only two children with obstructive hydrocephalus as a presenting feature of bacterial meningitis have been observed.^4^ The management of such cases has not been well described, and, here, we present the challenging management of this case.

## CASE REPORT

We present a case of a 16-week child who was presented to OPD of a rural health facility with complaints of multiple episodes of high-grade fever, that did not respond to antibiotics and antipyretics. The patient was referred to the Patan Academy of Health Sciences for further management. The child had a fever of 39.05°C on presentation. Blood investigations revealed seropositive for dengue virus infection with nonstructural protein 1 antigen (NS1) positive, IgG/ IgM negative with normal leukocyte count. He was admitted for observation. The child's medical history was unremarkable including a normal birth history, pregnancy, and delivery. The child was alert with soft fontanelles, moving all four limbs spontaneously, and bedside observations were within normal limits. Cardiorespiratory and abdominal examinations were unremarkable. There were no other abnormal clinical findings or signs of raised intracranial pressure.

The patient was afebrile by the end of the first week of admission but started to develop signs of meningeal irritation which include continuous crying, refusal of feeding, stiff neck on examination, and multiple episodes of vomiting during the course of admission.

The fever started to recur during the second week. The patient was on intravenous ceftriaxone and paracetamol. The head circumference of the child was monitored on a daily basis which showed gradual enlargement over a period of 2 weeks.

**Figure 1:**
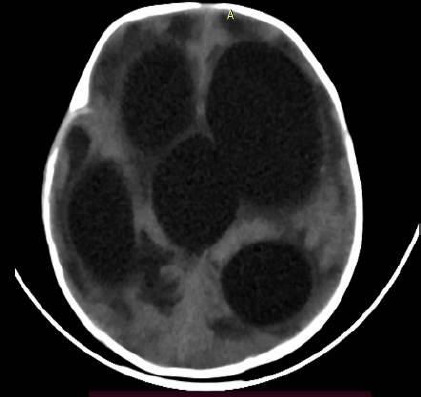
CT of the brain showing dilated ventricles.

Serial blood investigation showed a gradual increase in leukocyte count (18,000/mm^3^ at the end of the second week of admission). A computed tomography (CT) scan of the brain was done which showed enlarged bilateral ventricles and the third ventricle (Figure 1).

**Figure 2:**
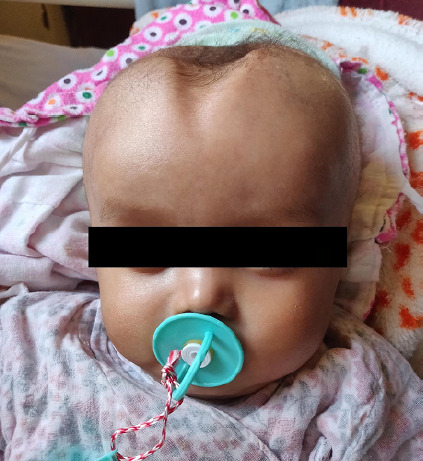
Depressed anterior fontanelle after VP shunting.

Cerebrospinal fluid (CSF) was sent for culture which revealed *E. coli* strain growth with sensitivity to piperacillin-tazobactam, imipenem and amikacin. The strain was, however, resistant to ceftriaxone and thus antibiotic was changed to piperacillin-tazobactam. CSF was grossly purulent on examination. Further analysis showed high protein content (465 g/dL), low levels of glucose (35 mg/dL), and a leukocyte count of 1600/mm^3^, all pointing towards bacterial aetiology. After new antibiotics were started, the fever started resolving with complete resolution by the end of the eighth week. However, the head size remained constant. Multiple CSF tappings were done but the head size remained enlarged with bulged fontanelle even after the eighth day after tapping. Median pressure ventriculoperitoneal (VP) shunting was planned and done on the 11th week. The patient was kept in PICU for observation following VP shunting. Head size decreased gradually and all other features of meningism also resolved (Figure 2).

The patient was then discharged on the third month of admission after normal findings in the CSF and blood investigations. On subsequent follow-up visits, the child was feeding well and had no features of meningism. Head size was also constant. All the neurological examinations were normal.

## DISCUSSION

Hydrocephalus can be defined as an active distension of the ventricular system of the brain resulting from the inadequate passage of CSF from its point of production within the cerebral ventricles to its point of absorption into the systemic circulation.^5^ An obstruction in the CSF channels leads to obstructive hydrocephalus. The foramen of Monro, the aqueduct of Sylvius, the fourth ventricle, and the foramen magnum are the places where obstructions happen most frequently. When CSF absorption at arachnoid granulations is impeded, it results in communicating hydrocephalus. Posthemorrhagic or post-inflammatory alterations are the most frequent causes. Meningitis, especially bacterial, can be complicated with hydrocephalus.^5^ Infants with hydrocephalus may have prenatal congenital conditions such as aqueductal stenosis or perinatal/ acquired causes like infection and bleeding. Rarely, acute bacterial meningitis can cause obstructive rather than communicating hydrocephalus.^6^

Ventriculitis is the inflammation of the ependymal lining of the cerebral ventricles, usually secondary to infection (for example meningitis, device-related or a complication of trauma). Early diagnosis is essential for appropriate treatment; this requires a clinical history, CSF sample, and imaging. Antibiotics are the mainstay of treatment. Due to the risk of recurrence and hydrocephalus, long-term follow-up is recommended. The ventricles and choroid plexus can serve as a reservoir of infection, even when the lumbar puncture yields sterile cultures.^7^

One series of unrecognized meningoventriculitis cases that presented hydrocephalus is highlighted in the literature review.^6^ Seventy-two paediatric patients were included in this retrospective study from India. Ten of the thirteen individuals with hydrocephalus who also had meningitis or ventriculitis first showed up with an expanding head circumference. All thirteen patients had risk factors, with ten of them necessitating postnatal antibiotics, including preterm birth, low birth weight, multiple births, neonatal sepsis symptoms, prolonged neonatal hospitalization, and perinatal antibiotic use. On CT imaging, aqueduct stenosis was present in nine cases.

Meningoventriculitis secondary to *E. coli* infection typically involves a fibro purulent inflammatory process as the chief cause of purulent newborn meningitis.^6^ Typically, an obstructive picture is noted to occur at the end of the second week when neutrophils degenerate and fibroblasts start to proliferate.^4^ Ventriculitis can result from the homogenous transmission of an infection through the glycogen-rich choroid plexus, which may promote local bacterial growth and serve as a reservoir for bacteria with relative resistance to antibiotics.^8,9^ In our patient, after the antibiotic therapy and clearance of *E. coli* which was confirmed by CSF culture, diversion of CSF was done by putting VP shunt.

Two similar cases have been described (one of whom died) in a study where the cause of obstructive hydrocephalus was later found to be *Streptococcus pneumoniae,* unlike our case.^4^

The case mentioned here illustrates how meningoventriculitis can cause infantile obstructive hydrocephalus. The treating clinician should constantly be on the lookout for potential meningitis causes while treating infantile hydrocephalus, and should always do a CSF culture to help chart a course of treatment.
